# Metabolic Regulation of Inflammation in Aging

**DOI:** 10.1093/geroni/igaf122.4162

**Published:** 2025-12-31

**Authors:** Michaella Niceforo, Gabrielle Chase, Lydia Gugliuzza, Ava Lankowski, Leena Bharath

**Affiliations:** Merrimack college, North Andover, Massachusetts, United States; Merrimack college, North Andover, Massachusetts, United States; Merrimack college, North Andover, Massachusetts, United States; Merrimack college, Merrimack College, Massachusetts, United States; Merrimack college, North Andover, Massachusetts, United States

## Abstract

Age-related cellular changes negatively impact the function of CD4+ T cells. Our prior work showed that mitochondrial complex II (Succinate dehydrogenase (SDH)) expression was upregulated in T cells from older (O) adults (60-80 years old). T cells from O adults also produced higher amounts of cytokines that are generally considered proinflammatory, such as Th17 cytokines, IL-17A/F, IL-21, and Th-17 supportive cytokine IL-6, compared to T cells from young (Y) adults (25-40 years old). The objective of our study is to evaluate whether hyperactivation of SDH is required for the induction of proinflammatory cytokines and the mechanistic link between SDH and Th17 cytokine production. CD4+ T cells were isolated from lean normoglycemic younger (Y; avg: 31.58 yrs; BMI 21.14 kg/m2) and older (O; avg: 64.81 yrs; BMI 21.95 kg/m2) adults. SDH was pharmacologically and genetically modulated, and mitochondrial structure, function, metabolites, and cytokine production were quantified. SDH activation in T cells from O adults induced heightened oxidation of succinate, disrupted the ratio of fumarate to succinate, stabilized HIF-1α, and promoted Th17 cytokines. Genetic and pharmacological inhibition of SDH in T cells from O adults lowered proinflammatory cytokine production, whereas exogenous addition of cell-permeable succinate induced SDH protein in T cells from Y adults, recapitulating the proinflammatory Th17 profile observed in T cells from O adults. These data establish a mechanistic link between SDH and Th17 inflammation.

